# Automated Identification of Hookahs (Waterpipes) on Instagram: An Application in Feature Extraction Using Convolutional Neural Network and Support Vector Machine Classification

**DOI:** 10.2196/10513

**Published:** 2018-11-21

**Authors:** Youshan Zhang, Jon-Patrick Allem, Jennifer Beth Unger, Tess Boley Cruz

**Affiliations:** 1 Department of Computer Science Lehigh University Bethlehem, PA United States; 2 Keck School of Medicine of USC Los Angeles, CA United States

**Keywords:** convolutional neural network, feature extraction, image classification, Instagram, social media, support vector machine

## Abstract

**Background:**

Instagram, with millions of posts per day, can be used to inform public health surveillance targets and policies. However, current research relying on image-based data often relies on hand coding of images, which is time-consuming and costly, ultimately limiting the scope of the study. Current best practices in automated image classification (eg, support vector machine (SVM), backpropagation neural network, and artificial neural network) are limited in their capacity to accurately distinguish between objects within images.

**Objective:**

This study aimed to demonstrate how a convolutional neural network (CNN) can be used to extract unique features within an image and how SVM can then be used to classify the image.

**Methods:**

Images of waterpipes or hookah (an emerging tobacco product possessing similar harms to that of cigarettes) were collected from Instagram and used in the analyses (N=840). A CNN was used to extract unique features from images identified to contain waterpipes. An SVM classifier was built to distinguish between images with and without waterpipes. Methods for image classification were then compared to show how a CNN+SVM classifier could improve accuracy.

**Results:**

As the number of validated training images increased, the total number of extracted features increased. In addition, as the number of features learned by the SVM classifier increased, the average level of accuracy increased. Overall, 99.5% (418/420) of images classified were correctly identified as either hookah or nonhookah images. This level of accuracy was an improvement over earlier methods that used SVM, CNN, or bag-of-features alone.

**Conclusions:**

A CNN extracts more features of images, allowing an SVM classifier to be better informed, resulting in higher accuracy compared with methods that extract fewer features. Future research can use this method to grow the scope of image-based studies. The methods presented here might help detect increases in the popularity of certain tobacco products over time on social media. By taking images of waterpipes from Instagram, we place our methods in a context that can be utilized to inform health researchers analyzing social media to understand user experience with emerging tobacco products and inform public health surveillance targets and policies.

## Introduction

Instagram, with millions of posts per day [1], can be used to inform public health surveillance targets and policies. However, this research relying on image-based data often relies on hand coding of images [2,3], which ultimately limits the scope of the study. Images from social media may be more useful than findings from text-based platforms alone (eg, Twitter and Reddit) when attempting to understand health behaviors, for example, user experiences with emerging tobacco products [4]. While automated image classification is useful for large-scale image classification (eg, processing and assigning labels to millions of images), current best practices in automated image classification are limited in their capacity to accurately distinguish between objects within images [5-7]. Automated image classification has been used in supervised, unsupervised, and hybrid approaches in classifying data [8-10]. Compared with unsupervised methods, supervised methods can be divided into stages of training and testing. The training stage consists of training a classifier by images and its labels, for example, describing image content, such as a person, dog, elephant, etc. The testing stage predicts the labels of the test images (in a new set of images) by a trained classifier.

Prior research has focused on ways to overcome the methodological challenges of automated image classification such as low accuracy. For example, Perronnin et al improved the Fisher Kernel approach to extend the bag-of-visual-words, also called bag-of-features (BOF), for large-scale image classification using internet images from ImageNet and Flickr, which increased precision from 47.9% to 58.3% but did not improve accuracy [5]. Verma et al used the backpropagation neural network approach to classify large images with good accuracy (97.02%), but this approach could not identify multiple categories of an image [6]. To reduce the time and spatial complexity of images, Simonyan et al proposed 2 visualization techniques using deep convolutional networks (ConvNets) to classify artificial images [7]. They combined understandable visualizations of ConvNets, maximizing the scores of images within different classes with gradient-based ConvNets visualization generating the saliency map (also called features map, which can represent the influence of pixels in image on image classification results) of every image (corresponding to one class) to use a deconvolution (also called transpose of convolution, which performs upsampling tasks instead of downsample in convolutional layer ) network to segment objects in the images [7].

These earlier approaches have moved automated image classification forward; however, there are still a number of significant limitations to overcome [11-13]. For example, the large number of images that need to be extracted to train a model requires great computational power. In addition, the BOF method cannot localize the objects within an image and cannot use visual word positions (eg, if a cup was in an image, BOF could not find its position) [14,15]. Support vector machine (SVM) have a limitation in showing the transparency of results, as the final model is difficult to visualize. Moreover, it is a challenge to choose a suitable kernel in kernel SVM [16-18]. A convolutional neural network (CNN), on the other hand, can improve the generalization of the algorithm and can solve nonlinear problems. While a CNN has high accuracy, to get better results, the parameters should be fine-tuned (eg, input image size, patch size, and the number of convolutional layers), and network performance is hard to optimize [19,20].

The purpose of this study is to determine whether combining CNN and SVM can achieve higher accuracy in image classification compared with CNN or SVM alone. To this end, data from Instagram containing images of waterpipes, also known as hookah (an emerging tobacco product possessing similar harms to that of cigarettes), were examined. By taking data from Instagram, we place our methods in a context that can be utilized to later inform researchers in the health domain who wish to analyze social media to understand user experience with emerging tobacco products and inform public health surveillance targets and public policies [2-4,21-25].

## Methods

### Data Acquisition

Data used in this study comprised posts on Instagram between February 19, 2016 and May 19, 2016, in the United States that included the hashtag #hookah. A total of 820 images was used in this study. The ground truth was manually labeled (hookah and nonhookah images). To balance the data and classes, the training images included 420 images (210 hookah and 210 nonhookah images), and test images also included 420 images (210 hookah and 210 nonhookah images). Further details on data collection are described elsewhere [24]. MATLAB was used to classify images into 2 categories: images containing a waterpipe (hookah) and those not containing.

### Convolutional Neural Network

**Figure 1:**
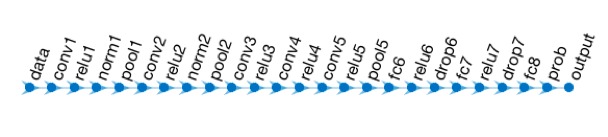
The architecture of AlexNet, which comprised 25 layers.

Image features comprising 25 layers were extracted using AlexNet [26-28] (a well-trained CNN software). Figure 1 shows the architecture of AlexNet. Among these 25 layers, there are input and output layers, 7 rectified linear units (ReLU) layers, 2 normalization layers, 3 pooling layers, 2 dropout layers (drop), 1 softmax layer (prob), and 8 learnable weights layers, which contain 5 convolutional layers (conv) and 3 fully connected layers (fc) [26]. The input layer comprised 227×227-pixel images. The ReLU layer reduces the number of epochs to achieve the training error rate higher than traditional tanh units. The normalization layer (norm) increases the generalization and reduces the error rate. The pooling layers summarize the outputs of adjacent pooling units [29]. The dropout layer efficiently decreases the test errors [30], and both dropout layer and the softmax layer reduce the overfitting phenomenon, while the output layer is the categories of images. To extract the features, we fine-tuned the network by removing the last 2 layers of the original 25 layers, as all layers are not suitable for extracting the features. As the layers at the beginning of the network can only detect the edges of the images, we used the results of the fully connected layers to extract features.

### Support Vector Machine

SVM, a supervised learning model with algorithms that analyze data for classification, has been used to predict the categories of objects in images [18,31]. Our proposed method goes beyond earlier research as the input (feature vectors) was based on the results of the CNN, which can boost accuracy and save time. AlexNet was used to extract features, and those features were used to then train the SVM classifier, requiring only minutes to train all images, thereby saving time [11]. Once the SVM classifier was trained using the feature vectors, the categories of images were predicted.

### Analytical Approach

**Figure 2:**
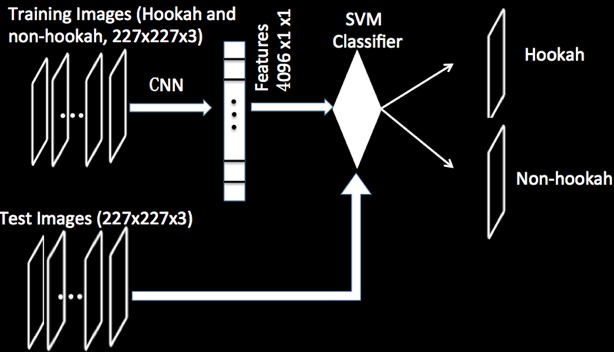
The scheme of our method. SVM: support vector machine; CNN: convolutional neural network.

**Figure 3:**
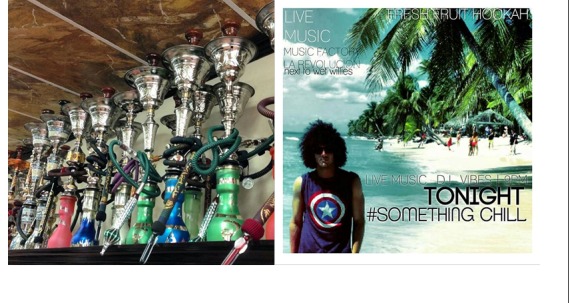
Examples of images with waterpipes (left) and without waterpipes (right).

First, we classified images into 2 categories—hookah and nonhookah images—and labeled accordingly. Figure 2 shows the classification scheme, for example, the input image dimension is 227×227×3 pixels, and the output of the CNN is the 4096×1×1 feature maps, which are used to train the SVM classifier; then, the classifier is used to predict the categories (hookah vs nonhookah) of test images. The hookah images contain a waterpipe, and the nonhookah images do not contain a waterpipe (Figure 3). Next, we divided image sets into training and test images; the training images were used to extract and learn the features (n=420, randomly selected), while the test images were used to calculate the accuracy of the method (n=420, randomly selected). To extract features of the images, the dimension of the input images was made uniform, for example, the image size was 227×227, as the image dimensions of 227×227 are the default of AlexNet. If an image is larger or smaller, we resized the dimensions of the input image to 227×227. We loaded the pretrained CNN by utilizing AlexNet [26], which has been trained by >1 million images. As discussed above, AlexNet was fine-tuned in our method, for example, we removed the last 2 layers of the AlexNet and used the data of the final fully connected layer. Based on the data of the last fully connected layer, we computed the features of the training and test images based on the CNN. Then, the class labels were extracted from the training and test image sets.

**Figure 4:**
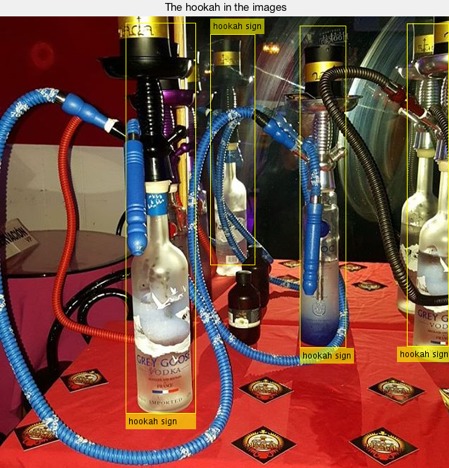
The localization of waterpipes in one image.

To optimize the SVM classifier, we automatically optimized hyperparameters (such as learning rate, the number of layers in the CNN, and mini-batch size) of the waterpipe features vector, and based on the optimized results, we arrived at an optimized SVM classifier (Figure 2) [18,32,33]. The input images dimension is 227×227×3. The output of the CNN was 4096×1×1 features maps of 2 image classes. These features were trained by the SVM classifier, and the trained classifier was later used to predict the categories of test images. We then assessed the performance of the SVM classifier by using the test images and increased the number of images to improve accuracy (the number of images increased from 42 to 420; Figures 3 and 4). Features of the waterpipes in the yellow box were extracted to train the SVM classifier. Based on the trained classifier, we predicted the classes of new images.

## Results

### Feature Extraction

**Figure 5:**
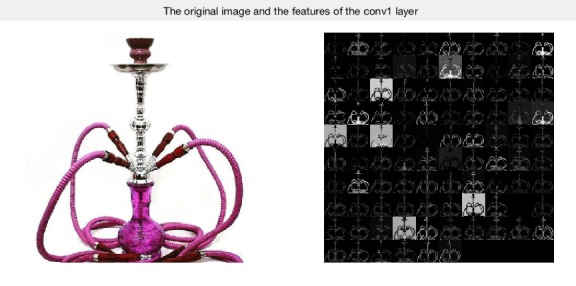
The extracted features of the first layer using the convolutional neural network.

**Figure 6:**
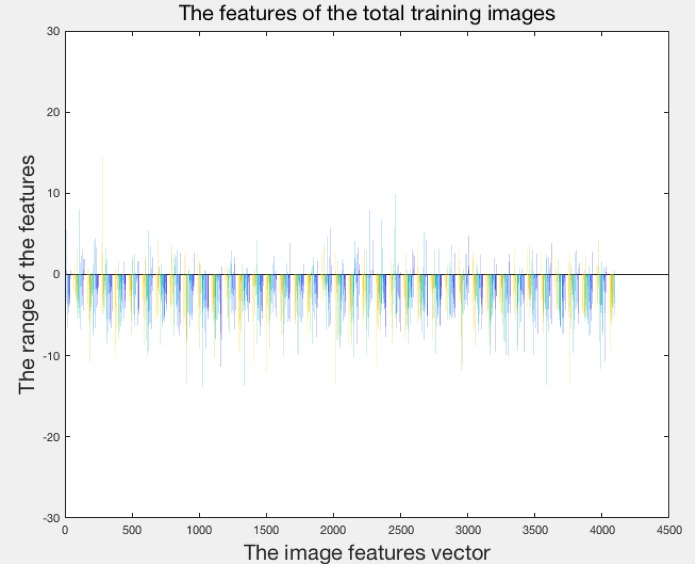
The features of the total image sets (420 images).

**Figure 7:**
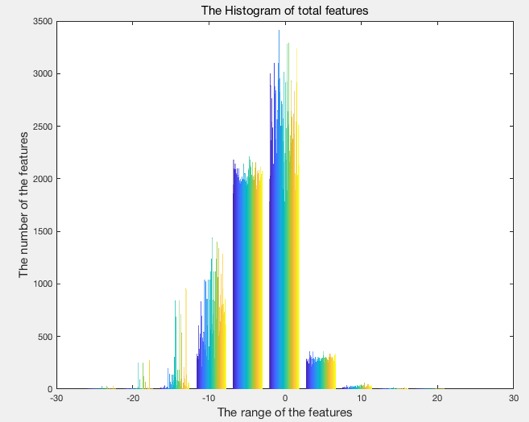
The histogram of the features. The interval of –2 and 2 contains the maximum number of features.

**Figure 8:**
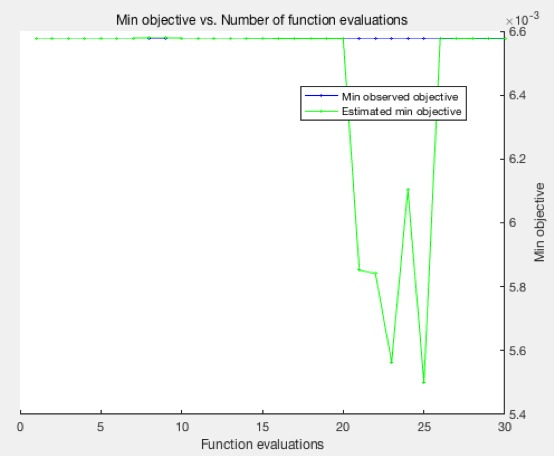
The relationship between the function evaluations and the minimum objective.

Results demonstrated that hookah features could be extracted by the CNN, with image categories classified by the SVM, maintaining a high level of accuracy: highest, 99.5% (418/420). Figure 5 shows the features that were extracted from the first convolutional layer; this layer can only detect the edges and blobs, while more features were extracted from the remaining convolutional layers. The original hookah image is on the left. The feature images (right) contains a montage of 96 images, which can reflect the processing of extracting features. Figure 6 shows the feature vectors of the 420 training images, with range –20 to 20; the majority of feature vectors are located between −10 and 10. The x-axis is the image features vector with 4096 total feature vectors. The y-axis is the range of the features with the range between –20 and 20. Figure 7 presents the histogram of the feature vectors. The maximum number of features was between –2 and 2; this interval reflected the most important features of the hookah images. Figure 8 shows the relationship between the function evaluations and the minimum objective. When the function evaluation was 25, the error between the minimum objective and the estimated minimum objective was the highest. Function evaluations demonstrated how many times to evaluate the optimized output. The minimum objective was the minimum observed value of the objective function; it is the smallest overall observation point if there are coupled constraints or evaluation errors.

The estimation of minimum objective functions can show the difference between the estimated (optimized) minimum objective and real minimum objective. The minimum objective and the estimated minimum objective are similar; however, there are differences across certain function evaluations. The maximum proportion of error is <.01, which is acceptable [19]. Based on the optimized SVM classifier, we evaluated the performance of our method by the test images.

### Test Image Classification Results

**Figure 9:**
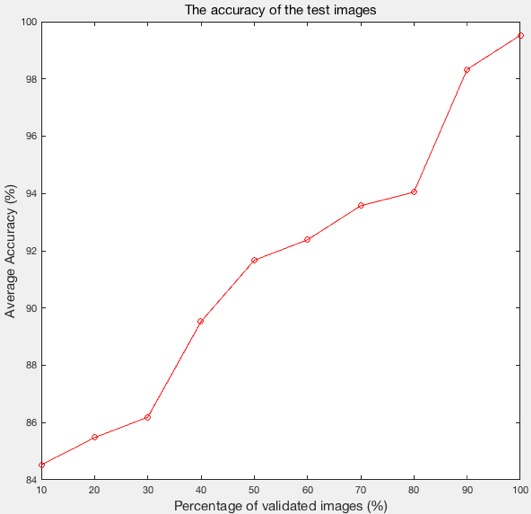
The learning curve showing the line graph of the accuracy of the classifier with a different number of validated images.

Figure 9 presents the learning curve showing the relationship between the percentage of validated images (eg, the training images, excluding the test images) and the average level of accuracy of the method. From the chart, with increase in the percentage of validated images, some of the accuracies boosted significantly. For example, from 80% (336/420), the accuracies increase faster than previous percentages, demonstrating that more training images are beneficial in predicting results. As the number of validated training images increased, the total number of extracted features increased: For 1 image, we can extract 4096 features; therefore, with the number of the validated training images (*n*) increased, the total number of extracted features can increase into *n* ×4096. In addition, as the number of features learned by the SVM classifier increased, the average level of accuracy increased. The number of validated images was equal to the percentage × the number of the training set; for example, if 10%, then the validated images=10%×420=42.

**Figure 10:**
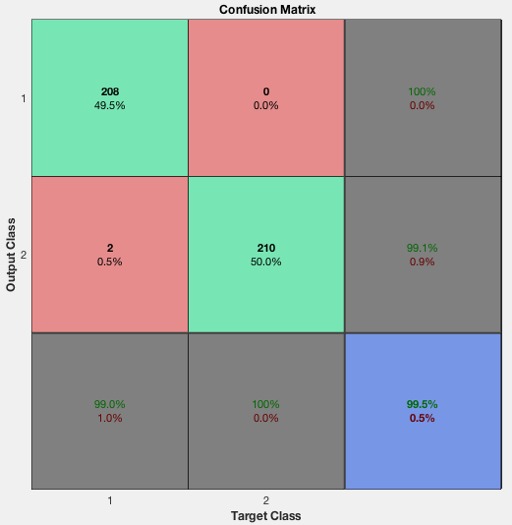
The confusion matrix of the test images (columns 1 and 2 are the hookah and nonhookah categories, respectively, column 3 is the accuracy of classified results).

Overall, 99.5% (418/420) of images classified were correctly identified as either hookah or nonhookah images (Figure 10). The first 2 green squares show the number of the test images and the percentage of the correct image classifications. For example, there were 208 images correctly classified as hookah, and this number accounted for 49.5% (208/420) of all test images. Similarly, 210 images were properly classified as nonhookah, and this accounted for 50% (210/420) of all test images. In the first row, all nonhookah images were correctly classified as such. In the second row, there were 2 hookah images incorrectly classified as nonhookah images, representing 0.5% (2/420) of all data. In the first row, 100% (208/208) of hookah images were correctly classified. In the second row, 99.1% (210/212) were correctly classified as hookah images. In the first column, 99% (208/210) were correctly classified as hookah images, and 0.1% (2/210) were correctly classified as nonhookah images. In the second column, out of 210 nonhookah images, 100% (210/210) were correctly classified as nonhookah images, and all images were correctly classified as hookah images.

### Comparison With Other Methods

**Figure 11:**
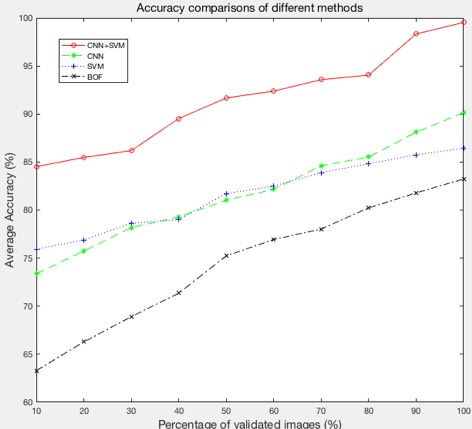
The prediction accuracy of different methods with different percentages of validated images. CNN: convolutional neural network; SVM: support vector machine; BOF: bag-of-features.

We compared our method with CNN, SVM, and BOF [12,34]. For SVM and BOF, the input was the original image (raw pixel values). Figure 11 shows how the accuracy of various models can be improved as a function of the size of the training data. Our method (CNN+SVM) had the highest accuracy, 99.5% (418/420), compared with other models (CNN, SVM, and BOF).

## Discussion

### Principal Findings

This study showed that the use of a CNN to extract features and SVM to classify images results in higher accuracy in automated image classification compared with using CNN or SVM alone. One crucial advantage of our pipelined approach is that we extracted sufficient features (4096 features from each image representing the details of each image) from a pretrained CNN model (AlexNet), taking advantage of SVM to train the features, saving time. Compared with earlier work using CNN, SVM, and BOF, our method improves accuracy when the number of training images is increased with accuracy reaching 99.5% (418/420), illustrating that our method is suitable for distinct images-like waterpipes.

The methods presented here could help detect increases in the popularity of certain tobacco products over time on social media. By identifying waterpipes in images from Instagram, we can identify Instagram users who may need tobacco-related education to curb hookah use. Instagram may be used to bolster the reach and delivery of health information that communicates the risk of hookah use [35-38]. Earlier research used Instagram images to capture and describe the context in which individuals use and are marketed tobacco products [3,24,25]. For example, the analysis of Instagram data on electronic cigarettes demonstrated that a majority of images were either individuals showing their favorite combinations of products (eg, type of electronic cigarette device and flavored juice) or people performing tricks with the products (eg, blowing a large aerosol cloud in competition with others) [25], demonstrating how and why people use this tobacco product. Previous analyses of hookah-related posts to social media websites provide information about hookah-related contexts, including the importance of stylized waterpipes, use of hookah in social settings, copromotion with alcohol [24], and primarily positive user experiences [39-41].

Earlier studies using image-based data provided timely information from a novel data source; however, their methods relied on hand coding of images—a process requiring time, expertise, and sample sizes small enough to reasonably code by hand, ultimately limiting the scope of the work. The findings from this study showed how automated image classification could be used to overcome such limitations. In addition, the methods from this study can help researchers in tobacco control identify what proportion of viewers on a social media site are interested in certain products; such methods may be crucial to document the every changing tobacco landscape.

### Limitations

The findings from this study should be considered with several limitations in mind, including the fact that our task was a simple binary classification (hookah vs nonhookah), which may result in high accuracy. To eliminate the problem of overfitting, we used ReLU, softmax, dropout layers in a CNN, and utilized several different training datasets (the number of datasets is different, which increased from 42 to 420; Figure 11). The methods developed in this study were only applied in the context of images from Instagram that focused on waterpipes and should be applied in more categories and other contexts in the future. While we had high accuracy in classification, accuracy could be improved with better input features from the CNN model. In the future, researchers should try to enlarge the sets of training images to extract specific features of an image, which may achieve higher accuracy with less computation power.

### Conclusions

Findings demonstrated that by combining CNN and SVM to classify images resulted in 99.5% (418/420) accuracy in image classification, which is an improvement over earlier method using SVM, CNN, or BOF alone. A CNN extracts more features of the images, allowing the SVM classifier to be better informed, which results in higher accuracy compared with methods that extract fewer features. Future research can use our method to reduce computational time in identifying objects in images.

## Abbreviations

BOF: bag-of-features
CNN: convolutional neural network
ConvNets: convolutional networks
FDA: Food and Drug Administration
ReLU: rectified linear units
SVM: support vector machine
